# Supplementary material for: Epigenetic specifications of host chromosome docking sites for latent Epstein-Barr virus
Source: Nat Commun. 2020 Feb 13;11:877. doi: 10.1038/s41467-019-14152-8 (PMC7018943; doi:10.1038/s41467-019-14152-8)
Supplement: Supplementary file 1 — Supplementary Information [file 41467_2019_14152_MOESM1_ESM.pdf]

Supplementary Information

Epigenetic Specifications of Host Chromosome Docking Sites for Latent Epstein-Barr Virus

Kyoung-Dong Kim[1], Hideki Tanizawa[2], Alessandra De Leo[3], Olga Vladimirova[3], Andrew Kossenkov[3], Fang Lu[3], Louise C. Showe[3], Ken-ichi Noma[2], and Paul M. Lieberman[3]*

[1]Department of Systems Biotechnology, Chung-Ang University, Anseong, Korea
[2]Institute of Molecular Biology, University of Oregon, Eugene, OR 97403, USA
[3]The Wistar Institute, 3601 Spruce Street, Philadelphia, PA 19146
*Corresponding Author
lieberman@wistar.org
(Phone) 215-898-9491

Supplementary Figure Legends

Supplementary Figure 1. Identification of total 4 peaks and reproducibility of datasets.

a Total number of 4C peaks for each bait for MutuI and Raji. **b** UCSC genome browser image showing 4C peaks from 5 baits at the chr2 (top panel) and chr2:143–164 Mb (bottom panel). **c** UCSC genome browser image showing that the series of 4C peaks with DS/Cp-1 baits at the indicated genomic region for the comparisons of biological replicates, restriction sites, and strains. **d**-**f** Pearson's correlation scores between biological replicates (Bio1 and Bio2, panel **d**), samples derived from two sides of a bait (MboI and Csp6I, panel **e**), and two strains (MutuI and Raji, panel **f**).

Supplementary Figure 2. Genome wide association between EBV and host chromosomes.

a Definition of Common 4C peaks. Common 4C peaks are defined as those that occurred as significant in at least 5 out of 6 total experimental datasets. **b** Representative example of common 4C peaks at the chr7: 144.5–148.5 Mb. Datasets are collected from DS/Cp-1 (Csp6I or MboI, Bio1 or Bio2) and DS/Cp-2 (Csp6I, Bio1 or Bio2). **c** Venn diagram showing co-occupancy between common 4C peaks for Raji and EBV-integration sites for Raji [49]. **d** Circos diagrams showing all significant association between EBV and host chromosomes. **e** Enlarged image of circos diagrams boxed in (panel **d**).

Supplementary Figure 3. 4C validation by FISH.

a Representative images of mitotic MutuI cells stained with dual FISH for EBV-488 and Target-594 (indicated genomic loci). Arrows indicate EBV signals on indicated chromosome. **b** Number of EBV dots on indicated chromosomes. Number of EBV dots were measured in more than 50 mitotic nuclei. *P*-value were calculated by two-sided Mann-Whitney *U* test. **c**, **d** Representative images of interphase MutuI (panel **c**) and Raji (panel **d**) nuclei stained with dual FISH for EBV-488 and Target-594. **e** Representative images of mitotic MutuI chromosomes stained with dual FISH for EBV-488 and Target-594.

Supplementary Figure 4. Effect of EBNA1 binding site effects on 4C peak enrichments.

a MutuI 4C peaks were analyzed for relative enrichment of RBP-jK, H3K9me3, EBF1, or 4C peaks for 4C target genes with EBNA1 binding sites (green) or without EBNA1 (non-binding) sites (grey). **b** EBNA1 binding sites are further enriched in DS/Cp common peaks that include overlapping peaks from the 4C dataset generated with FR primer relative to random genomic regions. Statistical analysis used a random sampling of 100 genes from each group (no gene was selected more than once) to calculate an average score. The random sampling was repeated 100 times and the distribution score was compared. Boxplots show center quartiles, midlines show medians and whiskers extend to the data points, which are no more than 1.5× the interquartile range from the box. *P*-value were calculated by two-sided Mann-Whitney *U* test.

Supplementary Figure 5. Correlation of EBV tethering sites between Mutu-LCL and GM12878.

a Total number of 4C peaks for each bait in Mutu-LCL (1 and 2). **b** Heatmap comparison of 4C in Mutu-LCL (DS/Cp-1/ MboI) with Hi-C in GM12878 and 4C in MutuI and Raji. **c** Average enrichment of 4C peaks (DS/Cp-1/ Csp6I) in Mutu-LCL, MutuI, and Raji and Hi-C peaks in GM12878 shown in panel **b**. The average enrichments are classified into 10 groups based on the enrichment of 4C peaks (Mutu-LCL/ DS/Cp-1/ MboI/ Bio1) scores (top: red → bottom: blue). **d** EBV copy numbers in cell lines used in this study, as determined by qPCR of total genomic DNA. **e** The 331 common 4C peaks for Mutu-LCL (1) (dark blue bars), 636 common 4C peaks for Mutu-LCL (2) (blue bars), and 568 Hi-C peaks for GM12878 (green bars) are loaded on human all chromosomes. **f** Example of 4C peaks for MutuI (DS/Cp-1/ Csp6I/ Bio1), Raji (DS/Cp-1/ Csp6I/ Bio1), and Hi-C peaks for GM12878 at the whole chromosome2 (top panel) and chr2: 82–97 Mb regions. **g** Table showing the number of overlapped peaks among Mutu-LCL (1), Mutu-LCL (2), GM12878, and MutuI.

Supplementary Figure 6. Different epigenetic features of EBV tethering sites according to latency types.

a-**c** Average ChIP-seq scores of the indicated proteins at the target loci of MutuI-4C (panel **a**), Raji-4C (panel **b**), and GM12878-Hi-C (panel **c**), over randomly selected loci were calculated. Two-sided t-test was performed and only *p*-values of below 0.001 are listed in this figure. The dashed line indicates the 2 times enrichment for target loci over random loci. The detailed is described in the **Experimental Procedures**.

Supplementary Figure 7. IPA canonical pathway analyses with EBV target genes.
a, **b** IPA canonical pathway analyses with 877 and 1,569 common 4C peaks for MutuI (panels **a-c**) and Raji (panel **d-f**), respectively. **a, d** All 4C peaks, **b, e** 4C peaks with EBNA1 binding sites, **c, f** 4C peaks lacking EBNA1 (non-binding) sites. The x-axis represents negative log *p*-values based on the probability that molecules in the datasets from EBV targets. Blue bars indicate overlap with panels **a** and **d**. Only the most significant pathways which are the threshold of significance for a *p*-value of 0.01 are shown. *P*-value were calculated by Fisher exact test.

Supplementary Figure 8. Validation of EBNA1 shRNA depletion studies.
a, b ChIP-qPCR for EBNA1 or control IgG in shEBNA1 or shCTRL transduced Raji cells for 4C target genes (panel **a**) or non-4C target genes (panel **b**). Error bar represents the standard deviation and *P*-value were calculated by two-sided Student *t*-test. *n*=3. **c, d** DNA FISH for EBV episomes in shCTRL or shEBNA1 transduced cells. Quantification for FISH signals shown as box plots (panel **c,** n> 50) and representative image (panel **d**). Boxplots show center quartiles, midlines show medians and whiskers extend to the data points, which are no more than 1.5× the interquartile range from the box. Dots indicate outliers. *P*-value were calculated by two-sided Student *t*-test. **e** 3C analysis of 4C peak at the chr2:15.6Mb location amplified by nested PCR and product confirmed by Sanger sequencing. **f** PCR analysis of EBV-host 3C product at chr2:15.6Mb (left) or Actin control (right) in Raji cells transduced with shCTRL or shEBNA1.

Supplementary Figure 9. Average Ct values for genes at 4C and control non-4C sites. Average Ct values for the RT-qPCR experiments as shown in Figure 6b and c. RT-qPCR analysis of mRNA expression in Raji cells transduced with shCTRL (blue) or shEBNA1 (orange) for EBNA1 or cellular top 10%-4C neuronal target genes or general

4C target genes (panel **a**) or non-4C target genes (panel **b**). Error bar represents the standard deviation. *n*=3.

List of Supplementary Tables

Supplementary Table 1. Sequence of adaptors.

Supplementary Table 2. Filtration of sequencing reads.

Supplementary Table 3. Number of sequencing reads according to adaptor categories.

Supplementary Table 4. Genomic position and sources of FISH probes.

Supplementary Table 5. Sources of ChIP-seq data.

Supplementary Table 6. IPA pathways for 4C target genes.

Supplementary Table 7. List of Tissue specific 4C target genes.

Supplementary Table 8. DNA oligonucleotide primers used for RT-qPCR, ChIP-qPCR, and 3C-PCR.

Supplementary Figures

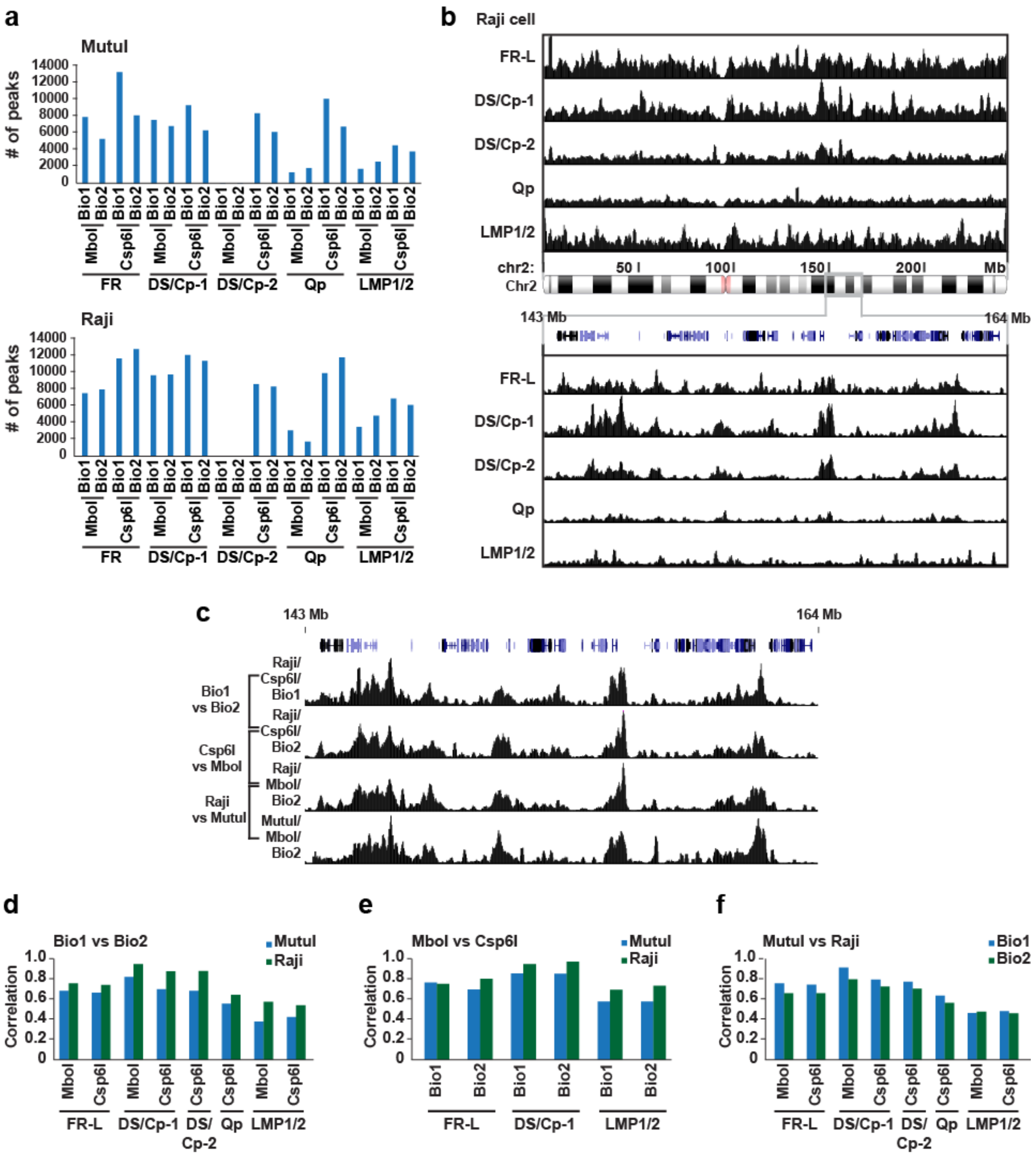

Supplementary Figure 1. Identification of total 4 peaks and reproducibility of datasets.

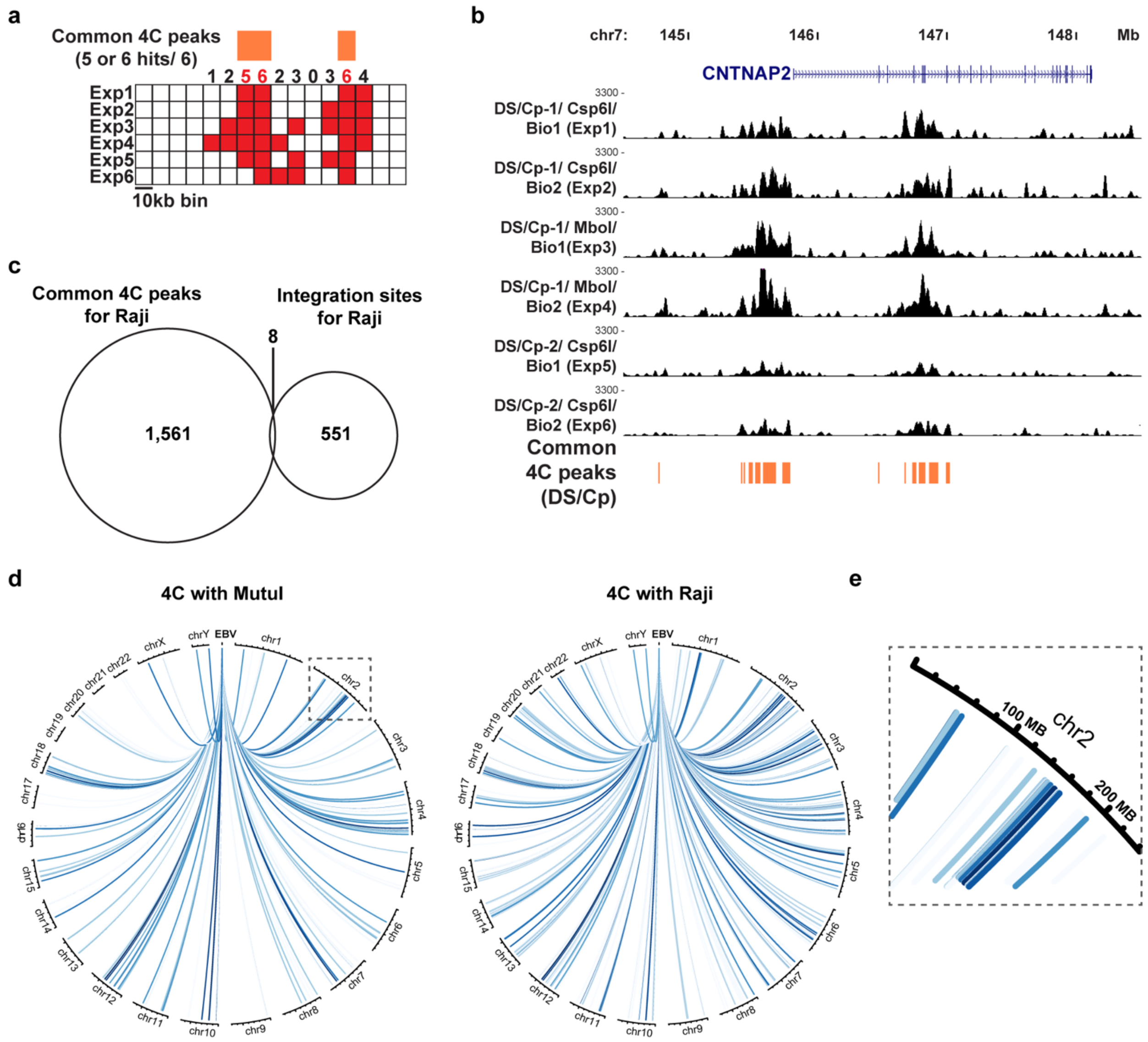

Supplementary Figure 2. Genome wide association between EBV and host chromosomes.

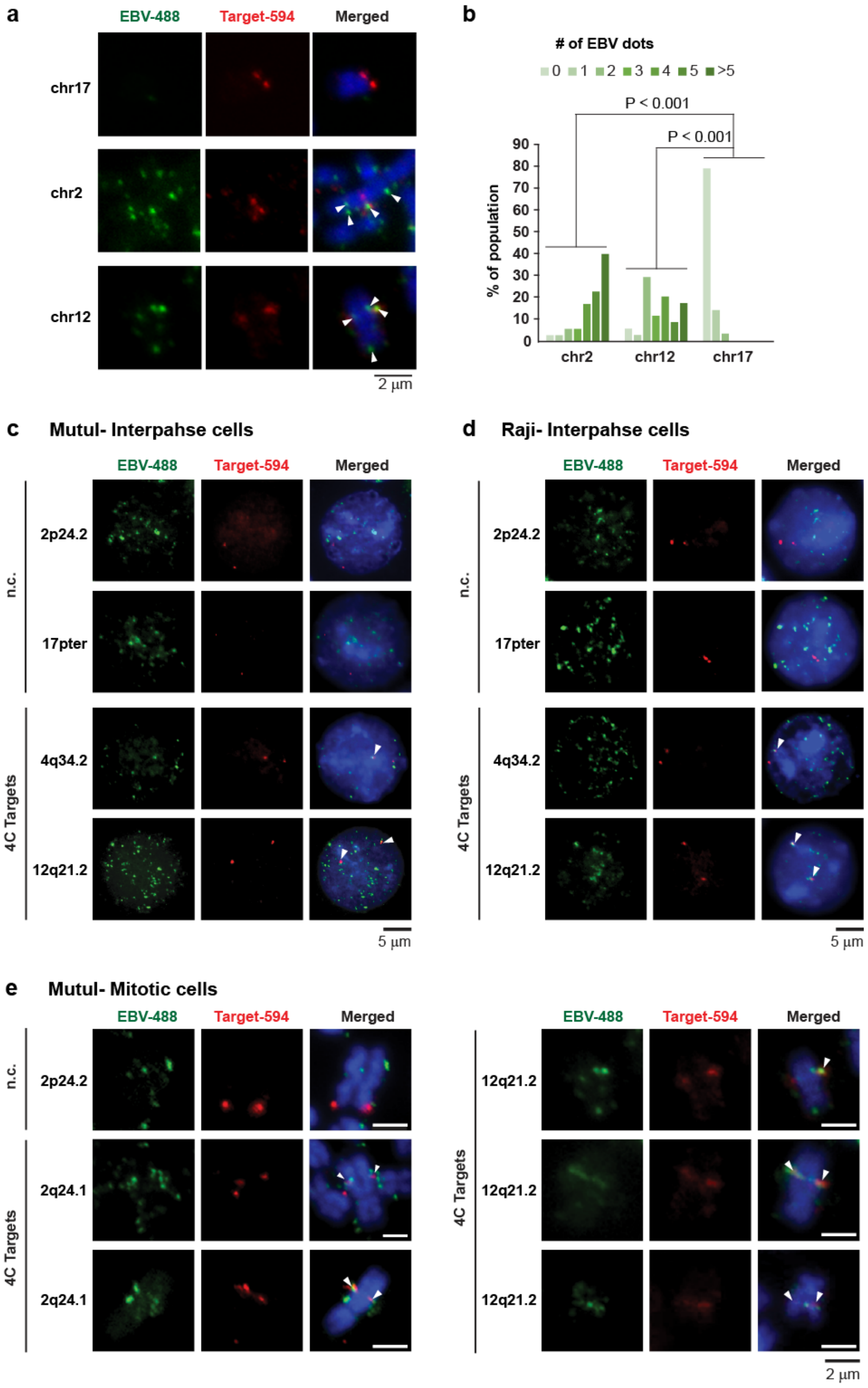

Supplementary Figure 3. 4C validation by FISH.

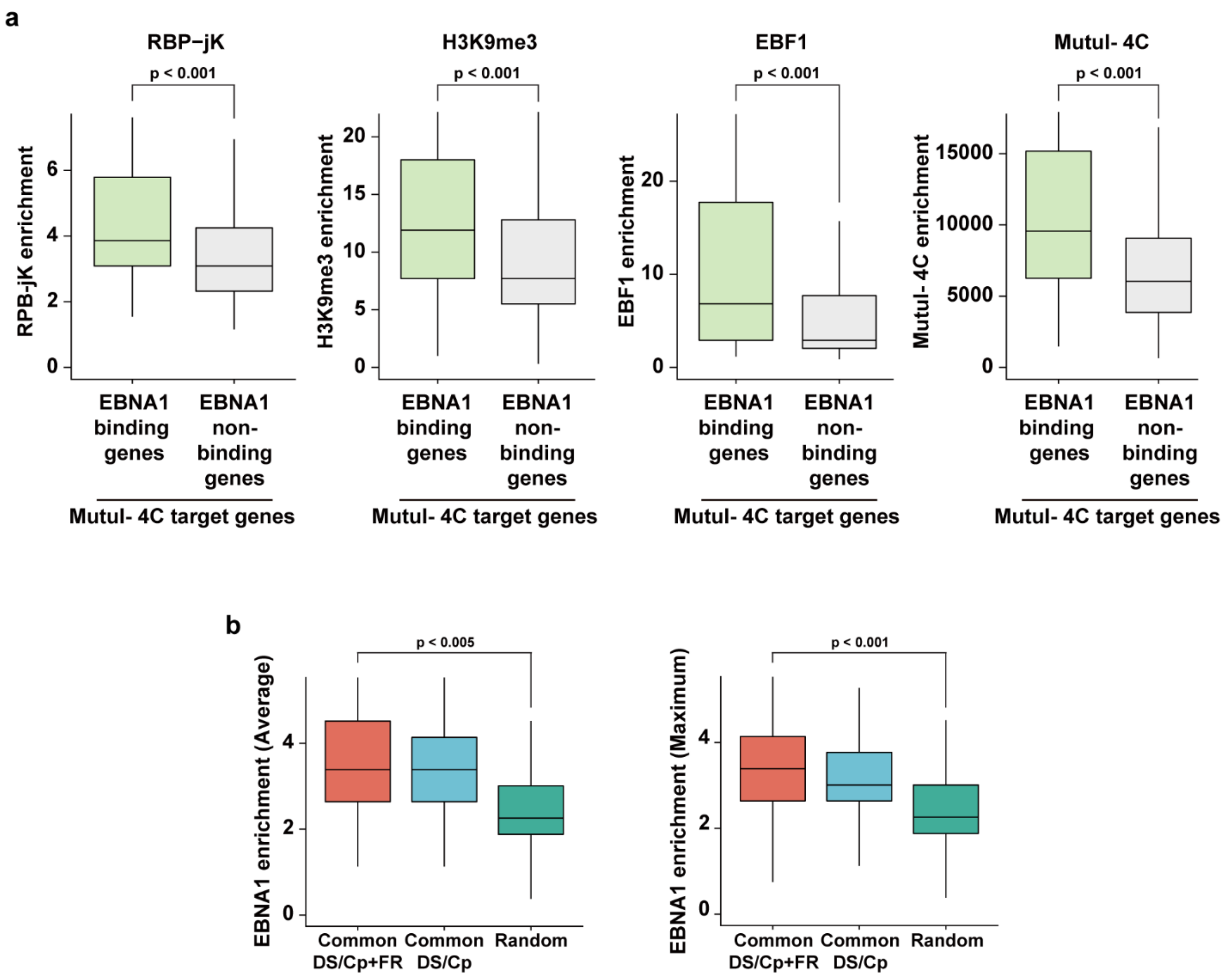

Supplementary Figure 4. Effect of EBNA1 binding site effects on 4C peak enrichments.

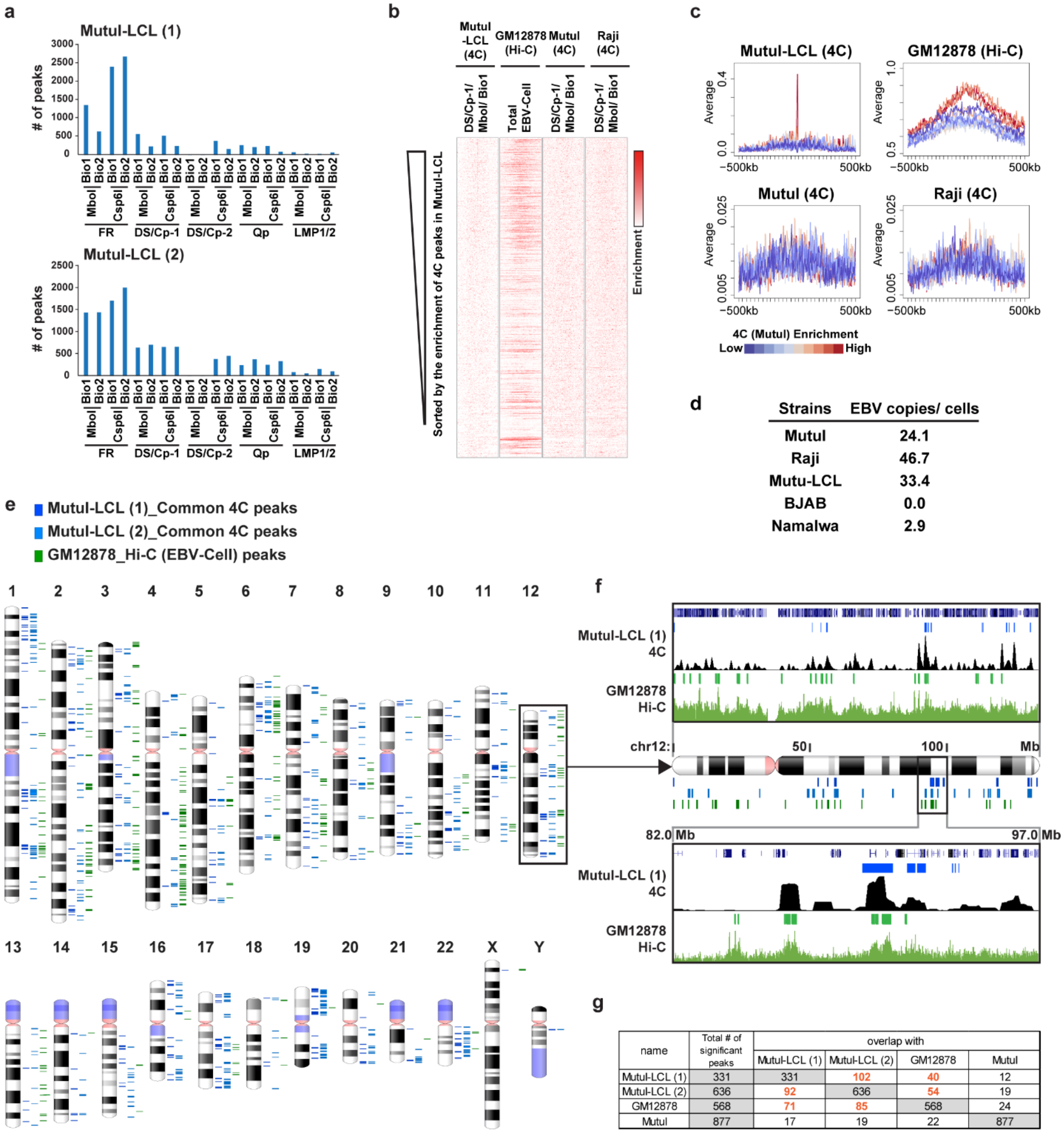

Strains	EBV copies/ cells
Mutul	24.1
Raji	46.7
Mutu-LCL	33.4
BJAB	0.0
Namalwa	2.9

name	Total # of significant peaks	overlap with			
		Mutul-LCL (1)	Mutul-LCL (2)	GM12878	Mutul
Mutul-LCL (1)	331	331	102	40	12
Mutul-LCL (2)	636	92	636	54	19
GM12878	568	71	85	568	24
Mutul	877	17	19	22	877

Supplementary Figure 5. Correlation of EBV tethering sites between Mutu-LCL and GM12878

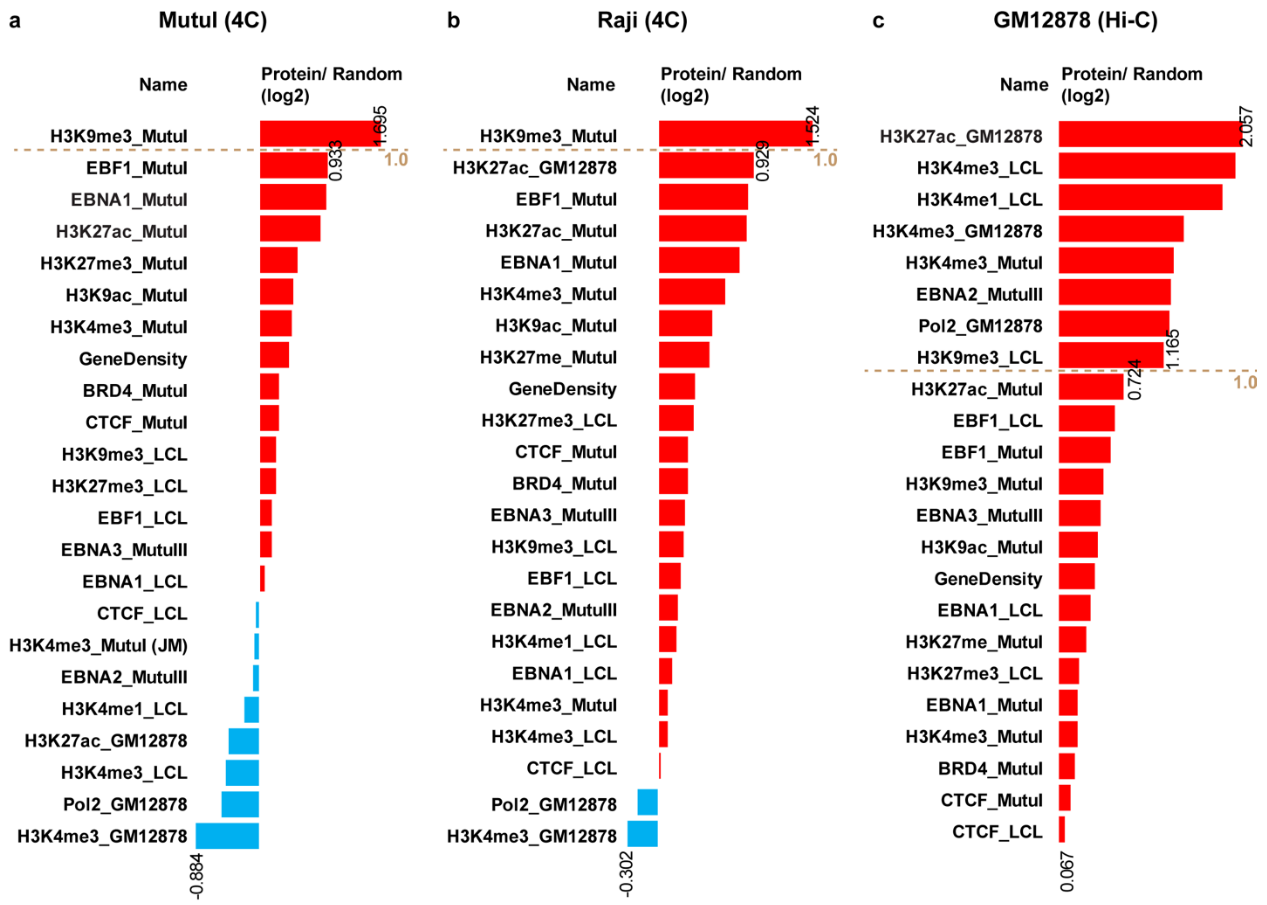

Supplementary Figure 6. Different epigenetic features of EBV tethering sites according to strains.

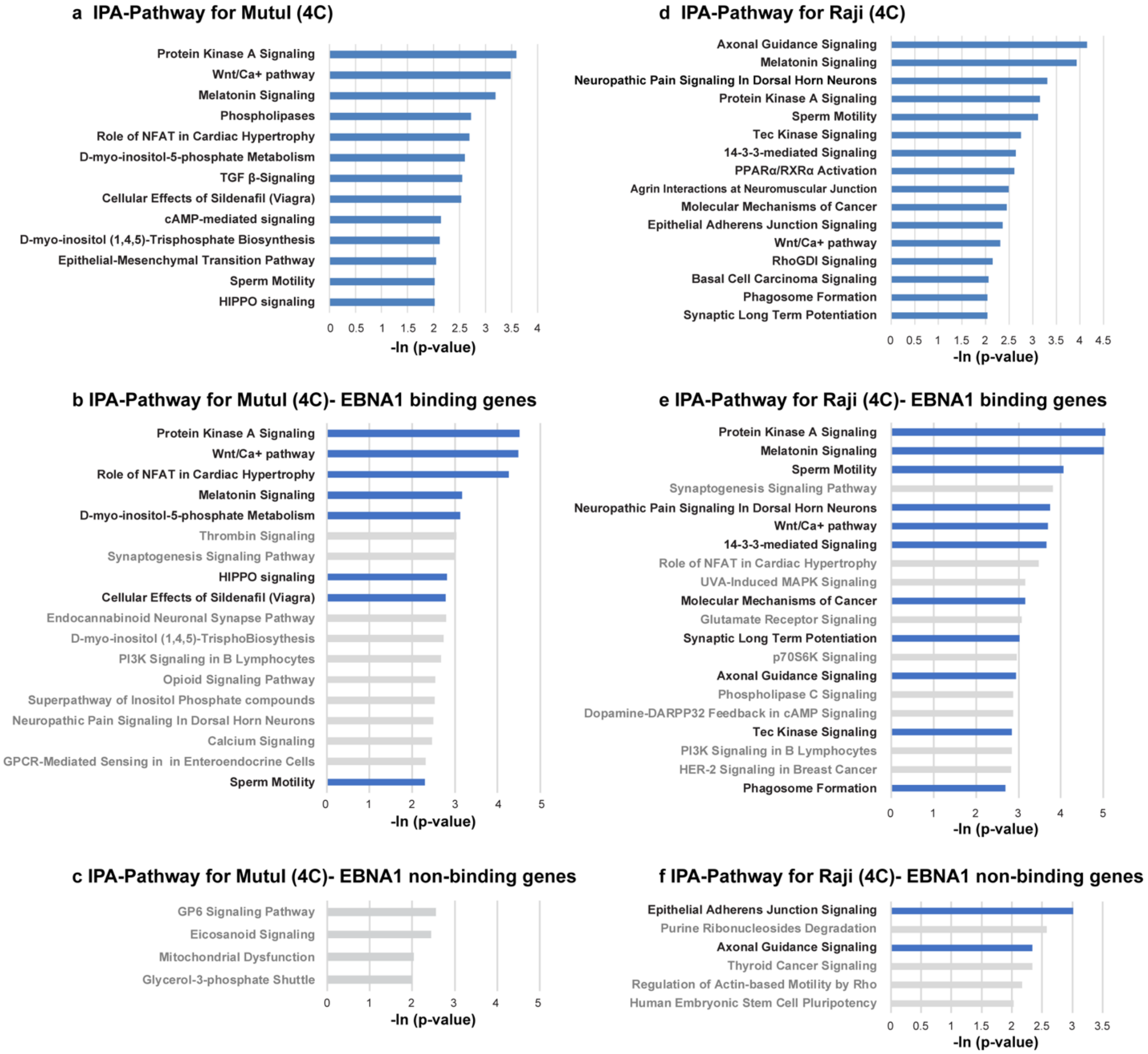

Supplementary Figure 7. IPA canonical pathway analyses with EBV target genes.

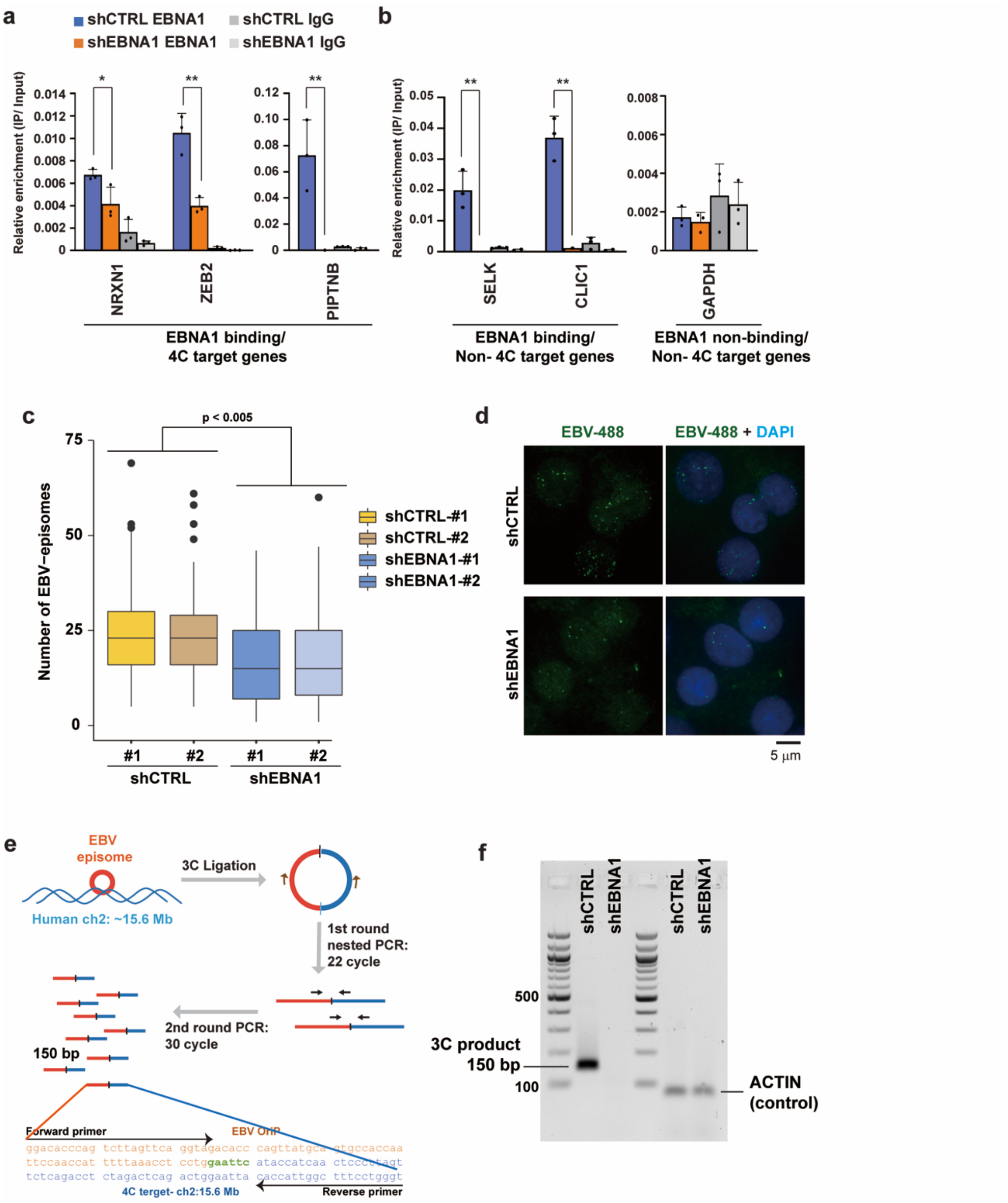

Supplementary Figure 8. Validation of EBNA1 shRNA depletion studies.

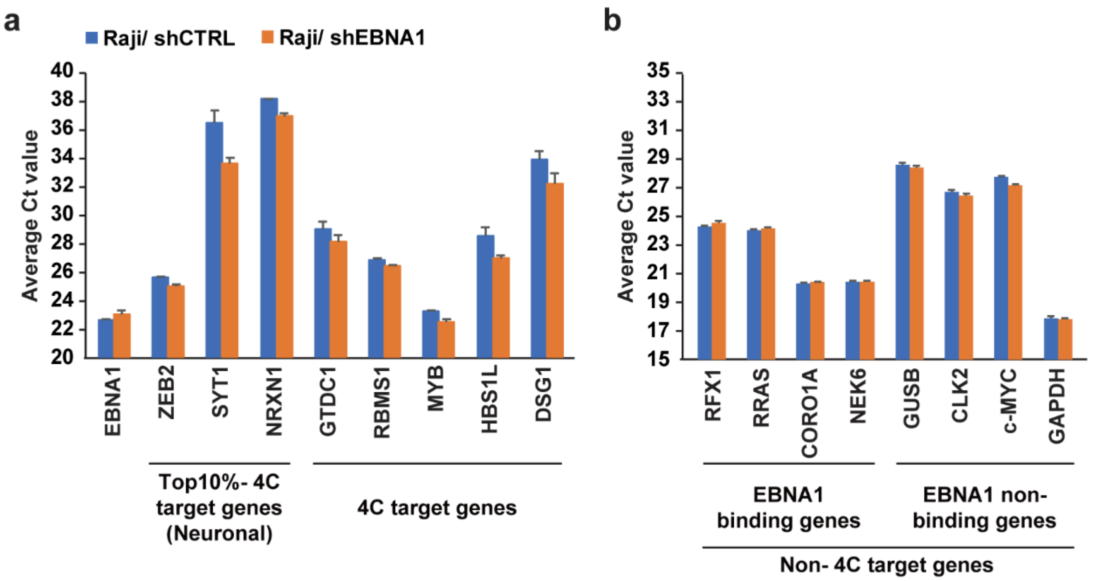

Supplementary Figure 9. Average Ct values for genes at 4C and control non-4C sites.

Supplemental Tables

Supplementary Table 1. Sequence of adaptors.

a List of Index primers.

Index primers	Replicates	Sequences
**UNIV**		AATGATACGGCGACCACCGAGATCTACACTCTTTCCCTACACGACGCTCTTCCGATCT
**Index01**	Bio1	CAAGCAGAAGACGGCATACGAGATCGTGATGTGACTGGAGTTCAGACGTGTGCT
**Index02**	Bio1	CAAGCAGAAGACGGCATACGAGATACATCGGTGACTGGAGTTCAGACGTGTGCT
**Index03**	Bio1	CAAGCAGAAGACGGCATACGAGATGCCTAAGTGACTGGAGTTCAGACGTGTGCT
**Index04**	Bio1	CAAGCAGAAGACGGCATACGAGATTGGTCAGTGACTGGAGTTCAGACGTGTGCT
**Index05**	Bio1	CAAGCAGAAGACGGCATACGAGATCACTGTGTGACTGGAGTTCAGACGTGTGCT
**Index06**	Bio2 for 01	CAAGCAGAAGACGGCATACGAGATATTGGCGTGACTGGAGTTCAGACGTGTGCT
**Index11**	Bio2 for 02	CAAGCAGAAGACGGCATACGAGATGTAGCCGTGACTGGAGTTCAGACGTGTGCT
**Index08**	Bio2 for 03	CAAGCAGAAGACGGCATACGAGATTCAAGTGTGACTGGAGTTCAGACGTGTGCT
**Index09**	Bio2 for 04	CAAGCAGAAGACGGCATACGAGATCTGATCGTGACTGGAGTTCAGACGTGTGCT
**Index10**	Bio2 for 05	CAAGCAGAAGACGGCATACGAGATAAGCTAGTGACTGGAGTTCAGACGTGTGCT

b List of view-point primers.

View-point primers	Sequences	Start	End	Orientation
**FR-MboI**	Univ-CGTGCATGGACCGGTTAATCCgatc	4731	4751	reverse
**FR-Csp6I**	Index-TCTTTCGCGAGGTTAGGGACAacacgttcctcgacaagtac	4941	4961	forward
**DS/Cp-1-MboI**	Univ-CCTGTGGCATGACTTCCTCCAGgtagaattggatc	9924	9945	reverse
**DS/Cp-1-Csp6I**	Index-GATTGCCTCTTGTGTTCTTGCCgcccaggtac	10281	10301	forward
**DS/Cp-2-MboI**	Univ-GGCATGACTTCCTCCAGGTAGAAttggatc	9918	9940	reverse
**DS/Cp-2-Csp6I**	Index-TTCTTGATTGCCTCTTGTGTTCTTGccgcccaggtac	10276	10300	forward
**Qp-MboI**	Univ-GGTGACAGGAGGGTCGACCTccggcgacctagtggtcccctccggatc	62278	62297	reverse
**Qp-Csp6I**	Index-ACGGTGTATGTTTGGGGACCCgcatgccggtac	62532	62552	forward
**LMP-MboI**	Univ-CCTCCTCCCAACGCGTTTCTgcggatc	167119	167138	forward
**LMP-Csp6I**	Index-GGCGCCCTTATTATTGATGTGACTtgtgatgcaataaataaaagtac	166959	166982	reverse

Supplementary Table 2. Filtration of sequencing reads.

Samples	MutuI_4C _bio1	Raji_4C_ bio1	Mutu-LCL (1) _4C_bio1	Mutu-LCL (2) _4C_bio1	MutuI_4C _bio2	Raji_4C_ bio2	Mutu-LCL (1) _4C_bio2	Mutu-LCL (2) _4C_bio2
**Total sequenced reads**	32,591,543	26,814,815	28,568,966	42,326,171	37,536,781	40,513,060	47,213,240	49,370,463
**Read with adapter**	24,637,686	22,802,656	17,624,881	25,248,030	25,186,519	26,522,102	23,180,355	24,898,777
**FR-MboI**	2,941,339	2,623,993	2,558,388	3,351,809	3,536,743	3,668,524	4,468,417	4,147,507
**FR-Csp6I**	3,039,404	2,808,613	2,552,120	3,204,651	3,507,778	3,531,879	4,050,743	3,776,244
**DS/Cp-1-MboI**	5,072,501	4,673,752	3,072,155	4,289,929	4,871,235	4,940,455	4,347,297	4,579,814
**DS/Cp-1-Csp6I**	2,844,792	2,751,985	1,917,992	2,677,197	2,759,271	2,735,832	2,924,785	2,812,972
**DS/Cp-2-MboI**	1,041	902	770	979	1,365	886	1,010	1,041
**DS/Cp-2-Csp6I**	1,875,300	1,850,422	1,120,207	1,577,783	1,745,085	1,902,184	1,307,922	1,665,035
**Qp-MboI**	2,391,812	2,309,718	2,053,204	2,879,505	2,554,977	3,271,824	2,408,357	2,597,255
**Qp-Csp6I**	2,316,411	2,189,573	2,005,954	2,915,934	2,303,584	2,979,057	2,089,787	2,203,706
**LMP-MboI**	1,899,822	1,514,019	1,118,822	2,157,538	1,839,131	1,721,169	889,242	1,699,753
**LMP-Csp6I**	2,255,264	2,079,679	1,225,269	2,192,705	2,067,350	1,770,292	692,795	1,415,450

Supplementary Table 3. Number of sequencing reads according to adaptor categories.

Name	Adaptor found read	Aligned read	Enough quality read	intra-chromosome (EBV)	inter-chromosome (Human)
**Mutul_FR_MboI_bio1**	2,941,339	2,092,001	1,774,421	1,280,326	494,095
**Mutul_FR_MboI_bio2**	3,536,743	2,395,394	1,835,741	1,051,156	784,585
**Mutul_FR_Csp6I_bio1**	3,039,404	2,764,777	2,494,339	1,332,156	1,162,183
**Mutul_FR_Csp6I_bio2**	3,507,778	3,206,341	2,855,127	1,324,748	1,530,379
**Mutul_DS/Cp-1_MboI_bio1**	5,072,501	4,893,388	4,560,537	3,817,730	742,807
**Mutul_DS/Cp-1_MboI_bio2**	4,871,235	4,656,449	4,059,289	2,627,032	1,432,257
**Mutul_DS/Cp-1_Csp6I_bio1**	2,844,792	2,690,485	2,513,164	1,746,225	766,939
**Mutul_DS/Cp-1_Csp6I_bio2**	2,759,271	2,579,534	2,332,997	1,176,802	1,156,195
**Mutul_DS/Cp-2_MboI_bio1**	1,041	420	351	278	73
**Mutul_DS/Cp-2_MboI_bio2**	1,365	762	643	467	176
**Mutul_DS/Cp-2_Csp6I_bio1**	1,875,300	1,784,968	1,664,324	1,170,807	493,517
**Mutul_DS/Cp-2_Csp6I_bio2**	1,745,085	1,642,269	1,478,442	752,810	725,632
**Mutul_Qp_MboI_bio1**	2,391,812	2,382,143	613,517	517,361	96,156
**Mutul_Qp_MboI_bio2**	2,554,977	2,541,464	629,875	505,825	124,050
**Mutul_Qp_Csp6I_bio1**	2,316,411	2,118,550	1,892,475	1,208,153	684,322
**Mutul_Qp_Csp6I_bio2**	2,303,584	2,115,362	1,905,852	1,237,021	668,831
**Mutul_LMP_MboI_bio1**	1,899,822	1,841,092	1,758,627	1,528,928	229,699
**Mutul_LMP_MboI_bio2**	1,839,131	1,776,448	1,645,067	1,283,244	361,823
**Mutul_LMP_Csp6I_bio1**	2,255,264	2,166,294	2,013,148	1,448,857	564,291
**Mutul_LMP_Csp6I_bio2**	2,067,350	1,954,413	1,748,125	975,533	772,592
**Raji_FR_MboI_bio1**	2,623,993	1,987,983	1,777,503	1,421,583	355,920
**Raji_FR_MboI_bio2**	3,668,524	2,842,580	1,929,705	1,555,553	374,152
**Raji_FR_Csp6I_bio1**	2,808,613	2,621,015	2,393,348	1,518,469	874,879
**Raji_FR_Csp6I_bio2**	3,531,879	3,244,194	2,987,581	2,214,125	773,456
**Raji_DS/Cp-1_MboI_bio1**	4,673,752	4,484,667	4,119,379	3,491,958	627,421
**Raji_DS/Cp-1_MboI_bio2**	4,940,455	4,388,476	3,795,971	3,161,894	634,077
**Raji_DS/Cp-1_Csp6I_bio1**	2,751,985	2,612,322	2,403,515	1,797,984	605,531
**Raji_DS/Cp-1_Csp6I_bio2**	2,735,832	2,463,002	2,111,058	1,562,479	548,579
**Raji_DS/Cp-2_MboI_bio1**	902	429	362	319	43
**Raji_DS/Cp-2_MboI_bio2**	886	363	306	247	59
**Raji_DS/Cp-2_Csp6I_bio1**	1,850,422	1,768,700	1,624,862	1,235,995	388,867
**Raji_DS/Cp-2_Csp6I_bio2**	1,902,184	1,738,545	1,485,544	1,099,044	386,500
**Raji_Qp_MboI_bio1**	2,309,718	2,299,915	719,252	636,873	82,379
**Raji_Qp_MboI_bio2**	3,271,824	3,239,273	920,398	807,072	113,326
**Raji_Qp_Csp6I_bio1**	2,189,573	1,980,718	1,779,318	1,358,304	421,014
**Raji_Qp_Csp6I_bio2**	2,979,057	2,678,287	2,383,090	2,058,118	324,972
**Raji_LMP_MboI_bio1**	1,514,019	1,447,947	1,359,780	1,142,817	216,963
**Raji_LMP_MboI_bio2**	1,721,169	1,641,980	1,544,412	1,341,847	202,565
**Raji_LMP_Csp6I_bio1**	2,079,679	1,985,732	1,829,057	1,309,343	519,714

**Raji_LMP_Csp6I_bio2**	1,770,292	1,676,686	1,539,991	1,149,887	390,104
**Mutu-LCL (1)_FR_MboI_bio1**	2,558,388	2,051,288	1,738,332	1,274,476	463,856
**Mutu-LCL (1)_FR_MboI_bio2**	4,468,417	3,782,065	3,059,107	2,268,019	791,088
**Mutu-LCL (1)_FR_Csp6I_bio1**	2,552,120	2,339,796	2,133,976	1,393,014	740,962
**Mutu-LCL (1)_FR_Csp6I_bio2**	4,050,743	3,693,496	3,339,525	2,361,291	978,234
**Mutu-LCL (1)_DS/Cp-1_MboI_bio1**	3,072,155	2,907,376	2,626,623	2,040,289	586,334
**Mutu-LCL (1)_DS/Cp-1_MboI_bio2**	4,347,297	4,080,448	3,706,274	2,974,613	731,661
**Mutu-LCL (1)_DS/Cp-1_Csp6I_bio1**	1,917,992	1,785,199	1,644,827	1,157,233	487,594
**Mutu-LCL (1)_DS/Cp-1_Csp6I_bio2**	2,924,785	2,696,124	2,474,722	1,857,297	617,425
**Mutu-LCL (1)_DS/Cp-2_MboI_bio1**	770	348	267	202	65
**Mutu-LCL (1)_DS/Cp-2_MboI_bio2**	1,010	546	446	384	62
**Mutu-LCL (1)_DS/Cp-2_Csp6I_bio1**	1,120,207	1,055,555	968,631	670,352	298,279
**Mutu-LCL (1)_DS/Cp-2_Csp6I_bio2**	1,307,922	1,222,068	1,124,746	838,407	286,339
**Mutu-LCL (1)_Qp_MboI_bio1**	2,053,204	2,043,135	560,871	480,761	80,110
**Mutu-LCL (1)_Qp_MboI_bio2**	2,408,357	2,384,323	852,957	745,682	107,275
**Mutu-LCL (1)_Qp_Csp6I_bio1**	2,005,954	1,788,646	1,636,061	1,282,809	353,252
**Mutu-LCL (1)_Qp_Csp6I_bio2**	2,089,787	1,854,048	1,674,596	1,347,302	327,294
**Mutu-LCL (1)_LMP_MboI_bio1**	1,118,822	1,071,929	1,015,529	862,204	153,325
**Mutu-LCL (1)_LMP_MboI_bio2**	889,242	826,879	770,412	656,575	113,837
**Mutu-LCL (1)_LMP_Csp6I_bio1**	1,225,269	1,161,054	1,066,705	858,541	208,164
**Mutu-LCL (1)_LMP_Csp6I_bio2**	692,795	641,459	573,733	479,735	93,998
**Mutu-LCL (2)_FR_MboI_bio1**	3,351,809	2,760,731	2,449,490	1,694,558	754,932
**Mutu-LCL (2)_FR_MboI_bio2**	4,147,507	3,541,300	2,814,158	2,168,448	645,710
**Mutu-LCL (2)_FR_Csp6I_bio1**	3,204,651	2,984,594	2,684,112	1,662,744	1,021,368
**Mutu-LCL (2)_FR_Csp6I_bio2**	3,776,244	3,467,030	3,149,631	2,387,643	761,988
**Mutu-LCL (2)_DS/Cp-1_MboI_bio1**	4,289,929	4,096,796	3,711,988	2,962,021	749,967
**Mutu-LCL (2)_DS/Cp-1_MboI_bio2**	4,579,814	4,216,369	3,778,764	3,182,477	596,287
**Mutu-LCL (2)_DS/Cp-1_Csp6I_bio1**	2,677,197	2,526,300	2,321,353	1,674,543	646,810
**Mutu-LCL (2)_DS/Cp-1_Csp6I_bio2**	2,812,972	2,609,288	2,371,357	1,873,864	497,493
**Mutu-LCL (2)_DS/Cp-2_MboI_bio1**	979	506	417	321	96
**Mutu-LCL (2)_DS/Cp-2_MboI_bio2**	1,041	496	394	343	51
**Mutu-LCL (2)_DS/Cp-2_Csp6I_bio1**	1,577,783	1,497,454	1,381,096	997,358	383,738
**Mutu-LCL (2)_DS/Cp-2_Csp6I_bio2**	1,665,035	1,556,101	1,423,505	1,128,959	294,546
**Mutu-LCL (2)_Qp_MboI_bio1**	2,879,505	2,868,532	738,569	630,684	107,885
**Mutu-LCL (2)_Qp_MboI_bio2**	2,597,255	2,572,522	930,583	827,522	103,061
**Mutu-LCL (2)_Qp_Csp6I_bio1**	2,915,934	2,637,579	2,395,539	1,879,355	516,184
**Mutu-LCL (2)_Qp_Csp6I_bio2**	2,203,706	1,958,690	1,737,684	1,463,134	274,550
**Mutu-LCL (2)_LMP_MboI_bio1**	2,157,538	2,074,259	1,963,884	1,696,830	267,054
**Mutu-LCL (2)_LMP_MboI_bio2**	1,699,753	1,598,883	1,492,403	1,300,603	191,800
**Mutu-LCL (2)_LMP_Csp6I_bio1**	2,192,705	2,100,811	1,928,911	1,549,045	379,866
**Mutu-LCL (2)_LMP_Csp6I_bio2**	1,415,450	1,349,020	1,251,582	1,024,259	227,323

Supplementary Table 4. Genomic position and sources of FISH probes.

Name	Position	NCBI CloneDB	Source
**2p24.2**	chr2:16,316,899-16,537,694	CH17-260N4	CHORI*
**2p16.3**	chr2:50,521,155-50,750,491	CH17-166I21	CHORI
**2q24.1**	chr2:154,707,246-154,925,297	CH17-339B17	CHORI
**2q24.2**	chr2:160,675,714-160,870,204	CH17-139O6	CHORI
**4q34.2**	chr4:175,422,993-175,620,206	CH17-330N20	CHORI
**12q21.2**	chr12:79,329,027-79,550,146	CH17-62J22	CHORI
**17pter**	chr17p subtelomeric region		Cytocell LTD

* CHORI: Children's Hospital Oakland Research Institute [https://bacpacresources.org]

Supplementary Table 5. Sources of ChIP-seq data.

ChIP-seq Data	GSM number
**H3K9me3_MutuI**	GSE129703
**CTCF_MutuI**	GSE129703
**EBNA1_MutuI**	GSM1905008
**H3K27me3_MutuI**	GSM1958046
**H3K4me3_MutuI**	GSM1958042
**EBF1_MutuI**	GSM1958038
**RPB-jk_MutuI**	GSM1958040
**BRD4_MutuI**	GSM2229482/ GSM2229483
**H3K27ac_MutuI**	GSM2229488/ GSM2229489
**H3K9ac_MutuI**	GSM2229490/ GSM2229491
**H3K4me3_MutuI**	GSM2229492/ GSM2229493
**H3K9me3_Mutu-LCL**	GSE129703
**CTCF_Mutu-LCL**	GSE129703
**H3K4me3_Mutu-LCL**	GSM1958043
**EBNA1_Mutu-LCL**	GSM1905009
**EBF1_Mutu-LCL**	GSM1958039
**RPB-jk_Mutu-LCL**	GSM1958041
**H3K4me3_GM12878**	GSM945188
**H3K27ac_GM12878**	GSM935386
**Pol2_GM12878**	GSM733771
**EBNA2_MutuIII**	GSM1153765
**EBNA3_MutuIII**	GSM1153766

Supplementary Table 6. IPA pathways for 4C target genes.

a IPA-canonical pathways for MutuI-4C target genes.

Canonical Pathways	*p*-value	Ratio	Number	Genes
**Protein Kinase A Signaling**	0.000257	0.0416	16	PLCB1, ADCY4, PTPRM, PTPRT, PDE4B, PLCH1, CAMK2A, UBASH3B, PTPN1, NFKB2, PTPRD, PDE7B, PTPN5, PLCG1, PTPN6, AKAP6
**Wnt/Ca+ pathway**	0.000331	0.0968	6	NFKB2, PLCB1, PLCG1, ROR1, PLCH1, CAMK2A
**Melatonin Signaling**	0.000646	0.0857	6	PLCB1, MAP2K3, PLCG1, RORC, PLCH1, CAMK2A
**Phospholipases**	0.001905	0.0847	5	PLCB1, PLCG1, PLCH1, LIPG, PLA2R1
**Role of NFAT in Cardiac Hypertrophy**	0.002042	0.045	10	PLCB1, ADCY4, CAMK1, CACNA2D1, MAP2K3, PLCG1, HDAC9, PLCH1, HDAC1, CAMK2A
**D-myo-inositol-5-phosphate Metabolism**	0.002512	0.0513	8	PLCB1, PTPRM, PPP1R1C, PLCG1, PTPN6, PLCH1, PPP1R16B, PTPN1
**TGF-β Signaling**	0.002818	0.0645	6	PITX2, MAP2K3, PMEPA1, HDAC1, ZFYVE9, BMPR1B
**Cellular Effects of Sildenafil (Viagra)**	0.002951	0.0556	7	PLCB1, ADCY4, PRKG2, MYH7B, PLCG1, PDE4B, PLCH1
**cAMP-mediated signaling**	0.007244	0.04	9	ADCY4, LTB4R, CAMK1, PDE7B, PDE4B, OPRM1, GPR17, AKAP6, CAMK2A
**D-myo-inositol (1, 4, 5)-Trisphosphate Biosynthesis**	0.007586	0.111	3	PLCB1, PLCG1, PLCH1
**Regulation of the Epithelial-Mesenchymal Transition Pathway**	0.008913	0.0415	8	NFKB2, WNT2, CDH12, ZEB2, MAP2K3, HMGA2, mir-8, PYGO1
**HIPPO signaling**	0.00955	0.0581	5	PARD3, ITCH, TEAD1, YAP1, FRMD6
**Sperm Motility**	0.00955	0.05	6	PLCB1, PRKG2, PLCG1, PDE4B, PLCH1, PLA2R1

b IPA-canonical pathways for Raji-4C target genes.

Canonical Pathways	*p*-value	Ratio	Number	Genes
**Axonal Guidance Signaling**	7.08E-05	0.0711	32	PDGFD, NFATC2, TUBB1, EPHA3, NTNG1, PRKACB, MAG, PTCH2, PAK5, ADAM29, WNT2, ARPC4, ITGB1, BDNF, PLCL1, BMP7, TUBA3C/TUBA3D, PLCB1, PLCD1, MME, GLI3, WASL, SRGAP1, PLCH1, UNC5C, PRKCD, PRKCA, NRP1, TUBB2B, ITGA4, SHC1, BMP3
**Melatonin Signaling**	0.000117	0.143	10	PLCB1, PLCD1, PRKCD, PRKCA, CAMK2D, MAP2K3, CAMK4, PRKACB, PLCH1, PLCL1
**Neuropathic Pain Signaling In Dorsal Horn Neurons**	0.00049	0.105	12	PLCB1, KCNN1, PLCD1, PRKCD, PRKCA, GRINA, CAMK2D, CAMK4, PRKACB, PLCH1, BDNF, PLCL1
**Protein Kinase A Signaling**	0.000708	0.0675	26	NFATC2, CAMK2D, PDE4B, PRKACB, PTCH2, PDE4D, PDE7B, PTPRF, SMPDL3A, PLCL1, AKAP6, PLCB1, PLCD1, PTPRM, EYA2, TCF4, GLI3, PTPRT, CAMK4, PLCH1, PTPN1, PRKCD, PRKCA, PTPRD, PTPRG, PTPRC
**Sperm Motility**	0.000776	0.1	12	PLCB1, PLCD1, PRKCD, PRKCA, PDE4D, CAMK4, PDE4B, PRKACB, PLCH1, PLCL1, FRK, PLA2R1
**Tec Kinase Signaling**	0.001778	0.0833	14	TNFSF12, MAPK10, JAK1, FRK, PAK5, PRKCD, PRKCA, ITGB1, ITK, BMX, TEC, ITGA4, RND3, VAV3
**14-3-3-mediated Signaling**	0.002291	0.0882	12	PLCB1, PLCD1, PRKCD, PRKCA, MAPK10, TUBB2B, TUBB1, YAP1, PLCH1, PLCL1, FOXO1, TUBA3C/TUBA3D
**PPARα/RXRα Activation**	0.002455	0.0805	14	PLCB1, PLCD1, ACVR2A, PRKACB, NR2C2, PLCH1, NCOA3, ACVR1B, PRKCA, NCOA6, MAP2K3, PLCL1, SHC1, GPD2

**Agrin Interactions at Neuromuscular Junction**	0.003236	0.11	8	ITGB1, MAPK10, NRG1, ITGA4, DVL1, PAK5, UTRN, LAMA2
**Molecular Mechanisms of Cancer**	0.003548	0.0619	24	PLCB1, ARHGEF3, TCF4, MAPK10, CAMK2D, JAK1, PRKACB, PTCH2, DVL1, PAK5, PRKCD, PRKCA, WNT2, ITGB1, BCL2, MAP2K3, ITGA4, BCL2L1, RND3, FOXO1, SHC1, CDK1, BMP7, BMP3
**Epithelial Adherens Junction Signaling**	0.004365	0.0816	12	PTPRM, PARD3, ARPC4, TCF4, TUBB2B, TUBB1, MYH7B, ACVR2A, MAGI1, WASL, ACVR1B, TUBA3C/TUBA3D
**Wnt/Ca+ pathway**	0.004898	0.113	7	PLCB1, PLCD1, NFATC2, PRKCA, PLCH1, DVL1, PLCL1
**RhoGDI Signaling**	0.007079	0.0739	13	ARHGEF3, CDH22, CDH12, WASL, PAK5, CDH18, PRKCA, DLC1, ARPC4, ITGB1, ITGA4, CDH11, RND3
**Basal Cell Carcinoma Signaling**	0.00871	0.101	7	WNT2, TCF4, GLI3, PTCH2, DVL1, BMP7, BMP3
**Synaptic Long-Term Potentiation**	0.00912	0.0813	10	PLCB1, PLCD1, PRKCD, PRKCA, GRINA, CAMK2D, CAMK4, PRKACB, PLCH1, PLCL1
**Phagosome Formation**	0.00912	0.0813	10	PLCB1, PLCD1, PRKCD, PRKCA, ITGB1, ITGA4, PLCH1, PLCL1, RND3, PLA2R1

Supplementary Table 7. List of Tissue specific 4C target genes.

Symbol	Description	Brain (z-score)	Nerve (z-score)	Adrenal Gland (z-score)
**CNTNAP4**	contactin associated protein-like 4	5.479	-0.176	-0.184
**SYT1**	synaptotagmin I	5.475	-0.159	-0.201
**SOX2-OT**	SOX2 overlapping transcript (non-protein coding)	5.473	0.091	-0.190
**RP11-275H4.1**	N.A.	5.428	0.572	-0.203
**CNTNAP2**	contactin associated protein-like 2	5.417	-0.247	-0.245
**NKAIN2**	Na+/K+ transporting ATPase interacting 2	5.388	0.820	-0.216
**RP11-4B14.3**	N.A.	5.366	0.880	-0.221
**KCNJ3**	potassium inwardly-rectifying channel, subfamily J, member 3	5.357	-0.179	-0.266
**GLRA3**	glycine receptor, alpha 3	5.288	-0.241	-0.247
**LINC00844**	long intergenic non-protein coding RNA 844	5.166	-0.023	-0.342
**EU233817**	disrupted in Rett 1 mRNA	5.151	-0.312	0.192
**PTPRD**	protein tyrosine phosphatase, receptor type, D	5.138	-0.380	-0.430
**PHYHIPL**	phytanoyl-CoA 2-hydroxylase interacting protein-like	4.937	-0.315	0.450
**NRXN1**	neurexin 1	4.914	2.081	-0.336
**LUZP2**	leucine zipper protein 2	4.893	-0.293	2.131
**TLL1**	tolloid-like 1	4.695	0.237	-0.539
**AC061961.2**	N.A.	4.482	-0.362	-0.362
**AC007560.1**	N.A.	4.150	2.040	-0.044
**SLC6A15**	solute carrier family 6 (neutral amino acid transporter), member 15	4.136	-0.303	-0.370
**PCDH11X**	protocadherin 11 X-linked	4.087	-0.272	-0.372
**FAM13C**	family with sequence similarity 13, member C	4.081	0.065	0.328
**SOX2**	SRY (sex determining region Y)-box 2	4.060	2.080	-0.465
**MTCO1P42**	MT-CO1 pseudogene 42	4.058	3.550	-0.254
**ZEB2**	zinc finger E-box binding homeobox 2	3.807	2.122	0.035
**NOL4**	nucleolar protein 4	3.792	-0.349	-0.359
**GTDC1**	glycosyltransferase-like domain containing 1	3.494	2.716	0.269
**RP11-597G23.1**	N.A.	3.162	-0.251	-0.251
**ZEB2-AS1**	ZEB2 antisense RNA 1	2.894	1.806	0.225
**NAV3**	neuron navigator 3	2.498	1.810	0.088
**RP11-720L8.1**	N.A.	2.181	-0.251	-0.251

**PYGO1**	pygopus homolog 1	2.049	0.164	-0.514
**MACROD2**	MACRO domain containing 2	2.005	-0.076	-0.339

Supplementary Table 8. DNA oligonucleotide primers used for RT-qPCR, ChIP-qPCR, and 3C-PCR.

a List of RT-qPCR primers.

Name of primers	Sequences
**EBNA1 RT-PCR FW**	GGTCGTGGACGTGGAGAAAA
**EBNA1 RT-PCR RV**	GGTGGAGACCCGGATGATG
**ZEB2 RT-PCR FW**	TCCTTTAAAAGAATCTTCCTTGTTCCTG
**ZEB2 RT-PCR RV**	GCATTTGAGAATTGTAGAAAGGCTTACT
**SYT1 RT-PCR FW**	ATCCAGAAAGTGCAGGTGGT
**SYT1 RT-PCR RV**	CAGCATGTCTGACCAGTGTC
**NRXN1 RT-PCR FW**	CAGGGATGGGTGGAACAGAT
**NRXN1 RT-PCR RV**	AAACCGTTGCCTCTCTCTCA
**GTDC1 RT-PCR FW**	ATAAAAATTACTCTGAATGAGTCTGGGC
**GTDC1 RT-PCR RV**	TCCCATGGAAGTGAGAAATGATTC
**RBMS1 RT-PCR FW**	CGGAACATACATGCCTGCAA
**RBMS1 RT-PCR RV**	TTGACCACTTGCCTCCTCAA
**MYB RT-PCR FW**	GGAGAGGTGGCATAACCACT
**MYB RT-PCR RV**	GACCTTCCGACGCATTGTAG
**HBS1L RT-PCR FW**	CAGCAAGAGAGACTGCACAC
**HBS1L RT-PCR RV**	TCCCACTTTCTGACAGCACT
**DSG1 RT-PCR FW**	GACCTGGTGTACTGCATCCT
**DSG1 RT-PCR RV**	CAGAGTGTCAGAGGTGGTGT
**RFX1 RT-PCR FW**	GAGATGCTGCGGGTGAAG
**RFX1 RT-PCR RV**	GGTGGTTGAGCGACGTGTA
**RRAS RT-PCR FW**	GGCCATGAGAGAGCAGTACAT
**RRAS RT-PCR RV**	GCCCACCTCGTTGAAACTC
**CORO1A RT-PCR FW**	TGTGGCCCTGATCTGTGAG
**CORO1A RT-PCR RV**	CATTCTTGTCCACACGTCCA
**NEK6 RT-PCR FW**	TGGGCTGTCTGCTGTACG
**NEK6 RT-PCR RV**	CACACTGCTCGATCTTCTGG
**GUSB RT-PCR FW**	CGCCCTGCCTATCTGTATTC
**GUSB RT-PCR RV**	TCCCCACAGGGAGTGTGTAG
**CLK2 RT-PCR FW**	CATTTAGCCGCTCATCTTCG
**CLK2 RT-PCR RV**	AGTCCCCGACGTGGTAGAT
**c-MYC RT-PCR FW**	GCTGCTTAGACGCTGGATTT
**c-MYC RT-PCR RV**	TAACGTTGAGGGGCATCG
**GAPDH RT-PCR FW**	CGGTGCGTGCCCAGTT
**GAPDH RT-PCR RV**	CTACTTTCTCCCCGCTTTTTTT

b List of ChIP-qPCR primers.

Name of primers	Sequences
**EBNA1bs_NRXN1 FW**	TCCTGGGGAACTGGATGGCA

**EBNA1bs_NRXN1 RV**	TCCCTAGCACTGGGCTCCTG
**EBNA1bs_ZEB2 RV**	ATTCCCGAGCTAGGGGCAGT
**EBNA1bs_ZEB2 RV**	TCCGCATGGGTTTTCTGGGT
**EBNA1bs_PITPNB FW**	TCTGGGCAGCCTACGCTTT
**EBNA1bs_PITPNB RV**	CGCAAAACGGCTTCCAAA
**EBNA1bs_SELK FW**	CGACGGGCGTTTTTGC
**EBNA1bs_SELK RV**	CCGCCTCCGCCTAACC
**EBNA1bs_CLIC1 FW**	CCTAAGCTGAGGGTGATTCATCTC
**EBNA1bs_CLIC1 RV**	CCCCACATCCTTGACAGGAA
**H3K9me3bs_NRXN1 FW**	AGGAGAATGAAAGGAAAAGAGGTGG
**H3K9me3bs_NRXN1 RV**	GGGATCACGTGTTTTTCCACTGC
**H3K9me3bs_DSG FW**	AGTGTAAGTCTCACGGACCCT
**H3K9me3bs_DSG RV**	ACAGAAGGTACTTGGTTGGCGG
**H3K9me3bs_ZEB2 FW**	TCTGGCTGCCATCAAGATGTTT
**H3K9me3bs_ZEB2 RV**	TGTCGAGTCACATGTCCAAGAAGC
**H3K9me3bs_SYT1 FW**	CTTGTCCAGAGATGAGGAGGAGT
**H3K9me3bs_SYT1 RV**	TGCATACCATTTACGCATTCTTAGACA
**H3K9me3bs_NAV3 FW**	TCAGCTAGTTGAATCAAACAGAGGAAA
**H3K9me3bs_NAV3 RV**	CTTCCTCATCTGACTCTTAAATGCCAA
**ACTIN FW**	GCCATGGTTGTGCCATTACA
**ACTIN RV**	GGCCAGGTTCTCTTTTTATTTCTG
**GAPDH FW**	TGGGCTACACTGAGCACCAG
**GAPDH RV**	GGGTGTCGCTGTTGAAGTCA

b List of 3C-PCR primers.

Name of primers	Sequences
**EBV_OriP_3C-EcoRI_outer primer**	AAGTCAGGATTCTCTAATCCCTCTG
**Chr2-156M_3C-EcoRI_outer primer**	ACCTAAGATTATGTCACCTGCAAAC
**EBV_OriP_3C-EcoRI_inner primer**	GGACACCCAGTCTTAGTTCAGGTAG
**Chr2-156M_3C-EcoRI_inner primer**	ACCCAGGAAAGCCAATGGTGT
